# The NEAT Domain-Containing Proteins of *Clostridium perfringens* Bind Heme

**DOI:** 10.1371/journal.pone.0162981

**Published:** 2016-09-16

**Authors:** Jocelyn M. Choo, Jackie K. Cheung, Jessica A. Wisniewski, David L. Steer, Dieter M. Bulach, Thomas J. Hiscox, Anjana Chakravorty, A. Ian Smith, David A. Gell, Julian I. Rood, Milena M. Awad

**Affiliations:** 1 Infection and Immunity Program, Biomedicine Discovery Institute and Department of Microbiology, Monash University, Clayton, VIC 3800, Australia; 2 Department of Biochemistry and Molecular Biology, Monash University, Clayton, Victoria, Australia; 3 Victorian Bioinformatics Consortium, Monash University, Clayton, Victoria, Australia; 4 School of Medicine, University of Tasmania, Hobart, Tasmania 7000, Australia; Montana State University Bozeman, UNITED STATES

## Abstract

The ability of a pathogenic bacterium to scavenge iron from its host is important for its growth and survival during an infection. Our studies on *C*. *perfringens* gas gangrene strain JIR325, a derivative of strain 13, showed that it is capable of utilizing both human hemoglobin and ferric chloride, but not human holo-transferrin, as an iron source for *in vitro* growth. Analysis of the *C*. *perfringens* strain 13 genome sequence identified a putative heme acquisition system encoded by an iron-regulated surface gene region that we have named the Cht (***C****lostridium perfringens* **h**eme **t**ransport) locus. This locus comprises eight genes that are co-transcribed and includes genes that encode NEAT domain-containing proteins (ChtD and ChtE) and a putative sortase (Srt). The ChtD, ChtE and Srt proteins were shown to be expressed in JIR325 cells grown under iron-limited conditions and were localized to the cell envelope. Moreover, the NEAT proteins, ChtD and ChtE, were found to bind heme. Both *chtDE* and *srt* mutants were constructed, but these mutants were not defective in hemoglobin or ferric chloride utilization. They were, however, attenuated for virulence when tested in a mouse myonecrosis model, although the virulence phenotype could not be restored *via* complementation and, as is common with such systems, secondary mutations were identified in these strains. In summary, this study provides evidence for the functional redundancies that occur in the heme transport pathways of this life threatening pathogen.

## Introduction

Many pathogenic bacteria require iron to replicate and successfully cause an infection in the mammalian host. Iron is used in many important biological processes including DNA synthesis, the generation of energy and protection against reactive oxygen species [1]. However, the availability of iron in the mammalian host is limited because most iron is sequestered within cells, in the form of ferritin (iron-storage protein) and heme, an iron-porphyrin complex. Furthermore, the remaining extracellular iron is bound to the iron-binding glycoproteins transferrin and lactoferrin [1, 2]. Heme is the most abundant iron source in mammals, with most heme bound to hemoglobin (Hb), an oxygen-carrying molecule found in red blood cells [3]. Pathogens have developed various mechanisms to scavenge iron and heme from these host proteins to enable their survival within the host.

The best characterized heme uptake system in Gram-positive bacteria is the Isd system of *Staphylococcus aureus* [4, 5]. This system is also conserved in other Gram-positive pathogens including *Bacillus anthracis* and *Listeria monocytogenes* [6–8]. The Isd system of *S*. *aureus* encodes four cell wall-anchored surface proteins (IsdA, IsdB, IsdC and IsdH), a sortase B enzyme (SrtB), a membrane transport system (IsdDEF) and two cytoplasmic heme oxygenases (IsdG and IsdI); the production of these proteins is upregulated under iron-depleted conditions [4, 6]. The cell wall-anchored proteins act as receptors that bind to heme or heme-containing proteins and extract heme. Heme is then transported across the cell membrane by the membrane transporter proteins. In the cytoplasm, heme oxygenases catalyze the degradation of heme to release free iron [5]. The heme binding activity of the IsdA, IsdB, IsdC and IsdH cell wall proteins of *S*. *aureus*, and the hemoprotein receptors of *B*. *anthracis*, *L*. *monocytogenes* and *Streptococcus pyogenes*, is performed by conserved near-iron transporter (NEAT) domain(s), which are bacterial-specific domains found in a collection of iron-regulated surface proteins [9–13]. These receptors contain between one to five copies of the NEAT domain, with each domain consisting of approximately 125 amino acids. NEAT proteins are important for bacterial growth, as well as for the establishment and persistence of infections caused by a variety of bacteria including *S*. *aureus*, *B*. *anthracis* and *S*. *pyogenes*, providing evidence for the importance of heme acquisition in the infection process [14–17].

In Gram-positive bacteria, many surface proteins are covalently anchored to the peptidoglycan layer by membrane-anchored sortases [18, 19]. Sortases are transpeptidases that cleave the C-terminal sorting signal of surface proteins to catalyze the formation of amide bonds with cross bridges of the peptidoglycan [20, 21]. The C-terminal sorting signal of surface proteins consists of an LPXTG or NPQTN motif for sortase recognition, a hydrophobic domain and a short charged tail. In *S*. *aureus*, Sortase A (SrtA) recognizes the LPXTG motif of the IsdA, IsdB and IsdH surface proteins. The *S*. *aureus isd* locus also encodes a second enzyme, sortase B, which recognizes the NPQTN motif of IsdC [22].

*Clostridium perfringens* is a Gram-positive, spore-forming anaerobic bacterium and is the causative agent of human gas gangrene or clostridial myonecrosis. This disease results from the contamination of a traumatic wound with vegetative cells or spores of *C*. *perfringens* type A [23]. The damaged, ischemic tissues surrounding the injury site facilitate spore germination and the growth of vegetative cells, which are in turn accompanied by the production of various extracellular toxins. Of these toxins, α-toxin has been shown to be essential for the disease process and works synergistically with another toxin, perfringolysin O, to result in fulminant clostridial myonecrosis [24, 25].

The host iron sources that can be utilized by *C*. *perfringens* are not known. However, sequence analysis of the *C*. *perfringens* strain 13 genome [26] has identified seven potential iron acquisition systems, including two heme acquisition systems, one ferrous iron acquisition system, three siderophore-mediated iron acquisition systems and one ferric citrate iron acquisition system [27]. One of the heme acquisition systems, which we have designated as Cht (***C***. *perfringens* **h**eme **t**ransport), potentially encodes two NEAT domain-containing cell surface proteins (ChtD and ChtE), a sortase (Srt) and an ABC membrane transport system. In this study, we demonstrated that the *cht* locus was expressed under iron-limited conditions and that the NEAT proteins ChtD and ChtE were involved in heme binding. Mutation of the *chtDE* or *srt* genes did not impair ferric chloride (FeCl_3_) or Hb utilization and although these mutants were attenuated for virulence, secondary mutations presumably were responsible for this phenotype. This study highlights the importance of iron acquisition in *C*. *perfringens* JIR325 since functionally redundant uptake systems were found to be encoded by this bacterium.

## Materials and Methods

### Ethics statement

All virulence trials in this study were approved by the Monash University Animal Ethics Committee (Ethics number: MARP/2011/093) and were conducted in accordance with the guidelines of the Victorian State Government legislation.

### Bacterial strains, plasmids and media

All bacterial strains used in this study are listed in Table 1. A *C*. *perfringens* strain 13 derivative, JIR325 [28], was used as the parent strain in this study. *C*. *perfringens* cultures were grown in tryptic soy broth (TSB) (Difco), fluid thioglycollate broth (FTG) (Difco) or on nutrient agar (2.5% (w/v) nutrient broth (Oxoid), 0.3% (w/v) yeast extract (Oxoid), 0.1% (w/v) sodium thioglycollate (Oxoid), 1.5% (w/v) agar (Oxoid)) [28]. Where necessary, the media were supplemented with the appropriate antibiotics: rifampicin (10 μg/mL), nalidixic acid (10 μg/mL), erythromycin (50 μg/mL) or thiamphenicol (10 μg/μL). Broth media were pre-reduced by boiling for 10 mins and then cooled to room temperature prior to inoculation. Agar cultures were grown at 37°C in 10% (v/v) H_2_, 10% (v/v) CO_2_ and 80% (v/v) N_2_. Antibiotics and chemical reagents were from Sigma, unless otherwise stated.

**Table 1:** Bacterial strains and plasmids used in the study of the *cht* locus.

Strains or plasmid	Characteristics	Reference
**Strains**		
JIR325	Rif^R^ Nal^R^ derivative of *C*. *perfringens* strain 13	[29]
JIR12755	JIR325 *ΔchtDchtE Ω cat(*P)	This study
JIR12773	JIR12755 Δ*cat*(P) *Ω ermBchtD*^*+*^*chtE*^*+*^	This study
JIR12614	JIR325 *srt*::*erm*(B)	This study
JIR12861	JIR12614 (pJIR4151)	This study
**Plasmids**		
pET28a(+)	*E*. *coli* expression vector, N-terminal 6x His-tag, Kan^R^	Novagen
pJIR750	*E*. *coli-C*. *perfringens* shuttle vector, Cm^R^	[30]
pJIR751	*E*. *coli-C*. *perfringens* shuttle vector, Em^R^	[30]
pJIR2715	Suicide vector base for *C*. *perfringens*, Em^R^	[31]
pJIR2783	Suicide vector base for *C*. *perfringens*, Cm^R^, Erm^*R*^	[32]
pJIR3562	Targetron vector base for *C*. *perfringens*, Cm^R^, Erm^*R*^	[33]
pJIR3566	Targetron vector base for *C*. *perfringens*, *lacZ’*, Cm^R^, Erm^*R*^	[33]
pJIR3924	pCR-BluntII-TOPO carrying 2.1 kb *chtE* downstream sequence, Kan^R^	This study
pJIR3926	pCR-BluntII-TOPO carrying 2 kb *chtD* upstream sequence, Kan^R^	This study
pJIR3928	pJIR2783 with 2 kb *Bam*HI-*Pst*I *chtD* upstream fragment, Cm^R^, Em^R^	This study
pJIR3929	pJIR3928 with 2.1 kb *Sac*I-*Xho*I *chtE* downstream fragment, Cm^R^, Em^R^	This study
pJIR4029	pJIR751 with 5.3 kb *Asp*718-*Sph*I *chtD* to 650 bp of *chtA* fragment, Em^R^	This study
pJIR4032	pJIR3926 with 1.4 kb *Pst*I-*Not*I *erm*(Q) cassette of pJIR2715, Kan^R^	This study
pJIR4038	pJIR3566 carrying 353 bp *Bam*HI/*Hin*dIII fragment targeted to *srt*, *lacZ’*, Cm^R^, Erm^*R*^	This study
pJIR4053	pCR-BluntII-TOPO carrying 5.4 kb *Sac*I-*Pvu*I *chtD* to 650 bp of *chtA* fragment from pJIR4029, Kan^R^	This study
pJIR4055	pJIR4053 with 3.5 kb *Sac*I *isdA* upstream sequence and *erm*(Q) cassette of pJIR4032, Kan^R^	This study
pJIR4151	pJIR750 with 5.2 kb *Asp*718-*Sph*I *chtD* to *chtA* gene fragment, Cm^R^	This study
pJIR4080	Expression vector, pET28a(+) with 2 kb *Nhe*I-*Xho*I *chtD* gene fragment, Kan^R^	This study
pJIR4075	Expression vector, pET28a(+) with 486 bp *Nde*I-*Bam*HI *chtE* gene fragment, Kan^R^	This study
pJIR4077	Expression vector, pET28a(+) with 477 bp *Nde*I-*Bam*HI *srt* gene fragment, Kan^R^	This study

*Escherichia coli* strains DH5α (Life Technologies) and One Shot^®^Top10 (Life Technologies) were used to facilitate the construction of plasmids, whilst *E*. *coli* strain BL21(DE3) C43 [34] was used for recombinant protein expression. *E*. *coli* cultures were grown in 2x YT agar/broth under aerobic conditions at 37°C, unless otherwise stated. Where necessary, the media were supplemented with erythromycin (150 μg/mL), kanamycin (50 μg/mL) or chloramphenicol (30 μg/mL).

### Computational analyses

The accession number for the strain 13 genome sequence is BA00016.3, with the *cht* locus located between coordinates 291056 and 300674. Sequence similarity searches were performed using the BLAST program (www.ncbi.nlm.nih.gov/BLAST/). Pairwise identity scores of the NEAT domain amino acid sequences were obtained using the Clustal Omega multiple sequence alignment program (www.ebi.ac.uk/Tools/msa/clustalo/).

### Preparation of human hemoglobin

Red blood cells were freshly collected from a volunteer donor and washed in 0.9% (w/v) NaCl with centrifugation at 1,000 × g. Following collection of the red blood cells, and at each step in the procedure, the sample was saturated with carbon monoxide to maintain the human carboxyhemoglobin (HbCO) state, which is resistant to auto-oxidation. Red blood cells were lysed under hypotonic conditions and the stroma removed by centrifugation following re-adjustment by NaCl to 0.9% (w/v). The hemolysate was dialyzed against 20 mM Tris.HCl, 0.5 mM ethylenediaminetetraacetic acid (EDTA), pH 8.2, at 4°C and loaded onto pre-equilibrated Q-Sepharose anion exchange resin (GE Healthcare). The column was developed with a gradient of 0–0.2 M NaCl over 15 column-volumes and peak fractions containing HbCO were pooled. To obtain human oxyhemoglobin (HbO_2_), aliquots of HbCO were stirred on ice in a stream of pure O_2_ under illumination using white-light CREE LEDs (Ay Up Lighting systems, Samford QLD) as a high intensity light source. Complete conversion to HbO_2_ was monitored by UV-visible spectroscopy. Final globin concentrations were determined by UV-visible absorbance using the molar absorption coefficients ε_419_ = 192 000 M^–1^ cm^–1^ at 419 nm for HbCO, and ε_415_ = 129 000 M^–1^ cm^–1^ at 415 nm for HbO_2_.

### Molecular and genetic techniques

*E*. *coli* plasmid DNA was isolated by using an alkaline lysis method as previously described [35] or a QIAgen Spin Miniprep kit according to the manufacturers’ instructions. Chemically competent *E*. *coli* cells were prepared as previously described [36], unless stated otherwise. Electrocompetent *C*. *perfringens* cells were prepared as previously described [37] and electroporated with an electric pulse of 1.8 kV, resistance of 200 Ω and capacitance of 25 μF using an ECM630 Electro Cell Manipulator (BTX^®^ Harvard Apparatus). *C*. *perfringens* genomic DNA was isolated as before [38].

Restriction endonuclease digestions were prepared according to the manufacturers’ instructions (Roche Diagnostics, New England Biolabs). DNA ligations, DNA modifications and PCR were carried out as previously described [39]. The concentrations of DNA and RNA were determined using a Nanodrop^®^ spectrophotometer (ThermoFisher Scientific). Nucleotide sequencing reactions were performed by Micromon (Monash University, Clayton, Australia) using the PRISM Big Dye Terminator Premix (Applied Biosystems) and the sequences analyzed using the ContigExpress^™^ software (Thermofisher Scientific). Oligonucleotide primers synthesized by Sigma-Aldrich. All plasmids and oligonucleotide primers used in this study are listed in S1 Table.

### Construction of *chtDE* and *srt* mutants

*C*. *perfringens chtDE* and *srt* mutants were constructed by allelic exchange and Targetron technology, respectively. To generate the *chtDE* deletion mutant, an *chtDE* suicide plasmid, pJIR3929, was constructed. A 2 kb fragment containing the region directly upstream of *chtD*, including the first 77 bp of *chtD*, was PCR amplified using primers JRP4422 and JRP4410 (S1 Table). The PCR fragment was cloned into the multiple cloning site of pCR-BluntII-TOPO to obtain pJIR3926. The cloned fragment was digested with *Bam*HI and *Pst*I, purified using the QIAquick Gel Extraction kit (Qiagen), and subcloned into the equivalent sites upstream of the chloramphenicol resistance cassette of pJIR2783, generating pJIR3928. In a separate reaction, a 2.1 kb fragment encompassing the region directly downstream of *chtE*, including the last 24 bp of *chtE*, was PCR amplified using primers JRP3991 and JRP3992 (S1 Table). This PCR fragment was cloned into the multiple cloning site of pCR-BluntII-TOPO to generate pJIR3924. The plasmid was digested with *Sac*I and *Xho*I, the 2.1 kb fragment was purified as before and sub-cloned into the equivalent sites downstream of the chloramphenicol resistance cassette of pJIR3928 to obtain pJIR3929. All recombinant plasmids were confirmed by restriction digestion and nucleotide sequencing. The plasmid pJIR3929 was used to transform the wild-type *C*. *perfringens* strain, JIR325. Chloramphenicol-resistant and erythromycin-susceptible transformants were isolated and confirmed by PCR and Southern blot hybridization. The resultant *chtDE* deletion mutant was designated, JIR12755.

To construct an *srt* Targetron mutant, the best intron insertion site (lowest p-value) predicted by the Intron Site Finder (T. Seemann, personal communications) was chosen and the corresponding primers JRP4763, JRP4764, JRP4765 and JRP3867 used to generate a 353 bp PCR product using pJIR3562 as the DNA template. The PCR fragment was cloned into the *Hin*dIII and *Bsr*GI sites of the Targetron plasmid pJIR3566, to generate pJIR4038. This vector was used to transform JIR325. Erythromycin-resistant and chloramphenicol-susceptible transformants were isolated and confirmed as before. One of the resulting *srt* mutants, JIR12614, was chosen for further studies.

### Complementation of *chtDE* and *srt* mutants

The *chtDE* mutant was complemented *in cis* by allelic exchange using a suicide vector, pJIR4055, which was designed to insert an *erm*(Q) cassette ligated to the *chtD chtE* coding sequence construct upstream of the promoter of the *chtR* gene. Briefly, the *erm*(Q) cassette of pJIR2715 was digested with *Pst*I/*Not*I and subcloned into the corresponding sites of pJIR3926. The resulting plasmid pJIR4032, contained the 2.1 kb fragment encompassing the region upstream of *chtD* followed by an *erm*(Q) cassette. In a separate reaction, a 3.7 kb fragment encompassing *chtD* and *chtE*, including 549 bp and 158 bp of the upstream and downstream sequences, respectively, was PCR amplified using primers JRP4903 and JRP4583. The PCR fragment was digested with *Asp*718/*Bam*HI and subcloned into pJIR751, to generate pJIR3996. A 2.2 kb region downstream of *chtE* was PCR amplified using primers JRP4901 and JRP3992, digested with *Xba*I/*Sph*I and ligated in-frame to the *C*. *perfringens chtDE* sequence of pJIR3996. The resulting plasmid, pJIR4029, contained a 5.3 kb fragment of the wild-type sequence from 549 bp upstream of *chtD* to 650 bp downstream of the start codon of *chtA*. This fragment was subcloned into the *Sac*I/*Pvu*I site of pCR-BluntII-TOPO to generate pJIR4053. Finally, the *Sac*I-digested fragment of pJIR4032 containing the cloned sequence was subcloned into pJIR4053. The resulting plasmid, pJIR4055, was confirmed by restriction digestion and DNA sequencing, and then used to generate the *in cis* complemented *chtDE* strain, JIR12773.

For complementation of the *srt* mutant *in trans*, an *Asp*718/*Sph*I-digested 5.2 kb fragment of pJIR4029 containing the sequence 548 bp upstream of *chtD* to *chtA* was cloned into an *E*. *coli-C*. *perfringens* shuttle vector, pJIR750. The *srt* complementation plasmid, pJIR4151, was used to transform the *srt* mutant to generate the *in trans srt* complemented strain, JIR12861.

### RT-PCR analysis

RT-PCR was used to analyze the transcription of genes in the *cht* locus. Total RNA was isolated from JIR325 cells grown to mid-logarithmic phase under iron-limited conditions (TSB containing 100 μM 2,2-dipyridyl). Reverse transcription (RT) was carried out on 2 μg of RNA using 45 units of the AMW Reverse Transcriptase (Promega) in a 20 μL reaction, according to the manufacturer’s instructions, with several modifications including the use of 0.5 μg of random primers (Promega) and incubation at 42°C for 1 h. The RT reaction was terminated by heating the sample at 99°C for 5 min. For all RT-PCR analysis, a no RT control reaction (NRT) was performed, in which the reverse transcriptase was replaced with an equal volume of nuclease-free water. PCR amplifications were performed on the RT or NRT reactions using Taq polymerase (NEB) and the primers listed in S1 Table. As PCR controls, the sample was replaced with either nuclease-free water (negative control (NC), or with JIR325 genomic DNA (positive control (DNA)).

### Growth assays

*C*. *perfringens* cultures grown for 6 h in FTG media were used to inoculate 20 ml of pre-reduced TSB broth. The following day, 100 ml of fresh pre-reduced TSB broth (normal conditions), as well as pre-reduced TSB broths containing 150 μM 2,2-dipyridyl (Sigma) (iron-depleted conditions) only or supplemented with 200 μM FeCl_3_ (Sigma), 40 μM commercial human Hb (Sigma), 50 μM HbCO, 25 μM and 50 μM HbO_2_ or 7 μM human transferrin (Merck) in the holo-form (iron-replete conditions) were inoculated to a final turbidity at 600 nm of 0.1. Following reconstitution of the commercial lyophilized Hb, analysis by UV-visible spectrophotometry revealed absorbance peaks at approximately 405, 500 and 620 nm; comparison with published data [39] suggested this material contained predominantly ferric heme (methemoglobin (metHb)). The appropriate iron sources were added after 1 h of growth under iron-depleted conditions. Cultures were incubated at 37°C and bacterial growth was monitored by measuring the turbidity at 600 nm at 1 h intervals for 8 h.

### Virulence studies in a mouse myonecrosis model

The virulence of the isogenic *C*. *perfringens* strains was assessed in six- to eight-week old female BALB/c mice (purchased from the Walter and Elisa Hall Institute, Melbourne, Victoria) as previously described [40]. Mice were injected in the right hind thigh muscle with 50 μL of culture (approximately 10^9^ CFU) and monitored at 30 min intervals for a total of 12 h post-infection for disease symptoms including limping, blackening of the thigh or footpad, and swelling of the footpad. Mice were euthanized by CO_2_ asphyxiation when they showed severe disease in any of the symptoms other than swelling of the thigh. Statistical comparisons were carried out using the Mantel-Cox test for survival curves and the Mann-Whitney U test for survival time using GraphPad Prism software (GraphPad Software Inc).

### Purification of recombinant proteins and antibody generation

ChtD, ChtE and Srt were purified as recombinant proteins containing an N-terminal His_6_-tag. The resultant proteins lacked the predicted signal sequence at the N-terminus and/or the transmembrane domain and charged C-terminus. The gene regions encoding amino acids 31–711 of ChtD, 31–191 of ChtE and 29–187 of Srt, were PCR amplified from genomic DNA of JIR325 using the specific primers listed in S1 Table. The primer pairs were flanked with *Nde*I/*Bam*HI or *Nhe*I/*Xho*I restriction sites, and the reverse primers for the *chtD* and *chtE* gene fragments contained a stop codon (TAA). The PCR product and pET28a(+) expression vector were digested with the appropriate restriction enzymes and ligated to generate pJIR4080 (His-ChtD), pJIR4075 (His-ChtE) and pJIR4077 (His-Srt). The cloned gene fragments were confirmed by sequence analysis.

The plasmids were used to transform *E*. *coli* BL21 DE3(C43) cells to kanamycin resistance, and were grown overnight at 37°C on 2x YT agar supplemented with kanamycin. The transformants were used to inoculate 10 mL of 2x YT broth with kanamycin and grown overnight at 37°C. Overnight cultures were then subcultured (1:100 volume ratio) into 500 ml of 2x YT broth and incubated at 37°C with shaking (220 rpm) until the turbidity at 600 nm reached 0.6. Then, 0.5 mM of IPTG (isopropyl-β-D-galactopyranoside) was added to induce protein expression and the cultures incubated for an additional 4 h at 37°C. Cells were harvested by centrifugation at 9,300 x *g* for 10 min, washed once in phosphate buffered saline (PBS) (137 mM NaCl, 2.7 mM KCl, 10 mM Na_2_HPO_4_, 2 mM KH_2_PO_4_, pH 7.4) and resuspended in lysis buffer (20 mM Tris-HCl, 0.3 M NaCl, 10% glycerol, 5 mM imidazole, pH 7.9). The cells were lysed using an EmulsiFlex-C5 cell disrupter (Avestin Inc). The cell lysate was centrifuged at 27,000 x *g* for 20 min and the supernatant, which contained soluble proteins, subjected to affinity chromatography using Talon^®^ resin (Clonetech Laboratories) pre-equilibrated with lysis buffer. After 2 h of incubation at 4°C, the resin was washed three times with 10 ml of lysis buffer to remove unbound proteins. His-tagged proteins were eluted from the resin with 5 ml aliquots of elution buffer (60 mM imidazole, 20 mM Tris-HCl, 0.3 M NaCl, 10% glycerol, pH 7.9) and dialyzed overnight against 1.5 L of Tris buffer (150 mM NaCl, 50 mM Tris, 0.5 mM EDTA, 20% (v/v) glycerol, pH 7.5) with constant stirring at 4°C. The concentrations of the purified proteins were measured using the Pierce bicinchoninic acid (BCA) protein assay according to the manufacturer’s instructions (ThermoFisher Scientific).

Polyclonal antibodies specific to ChtD, ChtE and Srt were raised in New Zealand white rabbits by immunization with a total of 2 mg of each purified protein at the Institute of Medical and Veterinary Science, South Australia. Western blotting was used to determine the specificity and the final dilution for the ChtD (1: 80,000), ChtE (1:50,000) and Srt (1:2,500) antibodies.

### SDS-PAGE and Western blotting

Proteins or cell lysates were separated on 10% (w/v) SDS-PAGE acrylamide gels and stained using Coomassie Brilliant Blue, or transferred onto a Whatman Protran nitrocellulose membrane at 100 V for 1 h using a mini Trans-Blot^®^ Electrophoretic Transfer Cell (BioRad). Membranes were blocked for 1 h with 5% (w/v) skim milk (Diploma, Australia) in TBS-Tween [5 mM Tris-HCl, 15 mM NaCl, pH 7.4, 0.05% (v/v) Tween20]. The membranes were then washed twice (15 min wash followed by a 5 min wash) in TBS-Tween, incubated with a primary antibody in TBS-Tween for 1 h, washed again as before, incubated with a secondary antibody conjugated to horseradish peroxidase and washed as before. All incubations and washes were performed with shaking at room temperature. Membranes were developed with an enhanced chemiluminescent detection reagent (Amersham Life Science) in a FPM-100A film developer (FujiFilm).

### Heme association using absorbance spectroscopy

*E*. *coli* cells carrying the plasmids encoding the His-ChtD, His-ChtE or His-Srt proteins were grown in 1 L of 2x YT broth supplemented with kanamycin as before and induced for 17 h at 30°C. Each protein was purified as before and the His-ChtD (10 μM), His-ChtE (30 μM) and His-Srt (10 μM) proteins were diluted in Tris buffer. A hemin control sample was prepared by diluting 1 mg/mL of (bovine) hemin solution in Tris buffer (pH7.5) to a concentration of 10 μM. The absorbance of each sample between the wavelengths 250–650 nm was measured using a Cary^®^ UV-Visible spectrophotometer (Agilent^®^) and normalized using Tris buffer as a reference.

### Heme detection by MALDI TOF analysis

Protein samples were diluted in 50% (v/v) acetonitrile-0.1% (v/v) trifluoroacetic acid (TFA) solution in a 1:10 ratio. The sample solution was mixed in a 1:1 ratio with a matrix solution of 10 mg/mL α-cyano-4-hydroxycinnamic acid (Laser Biolabs) in TFA solution. The mixture was spotted onto a matrix-assisted laser desorption ionization (MALDI) target plate. The mass spectrometry and tandem time-of-flight (TOF/TOF) spectra were determined using a 4700 Proteomics Analyzer (Applied Biosystems) with a mass range of 500 to 3000 Da and focus mass of 1200 Da at 1500 shots per spectra. The spectra were processed using the 4000 series explorer software version 3.0 and calibrated using the plate method calibration against the 4700 mix peptide standards (Applied Biosystems).

## Results

### *C*. *perfringens* utilizes ferric chloride and human hemoglobin

**Fig 1:**
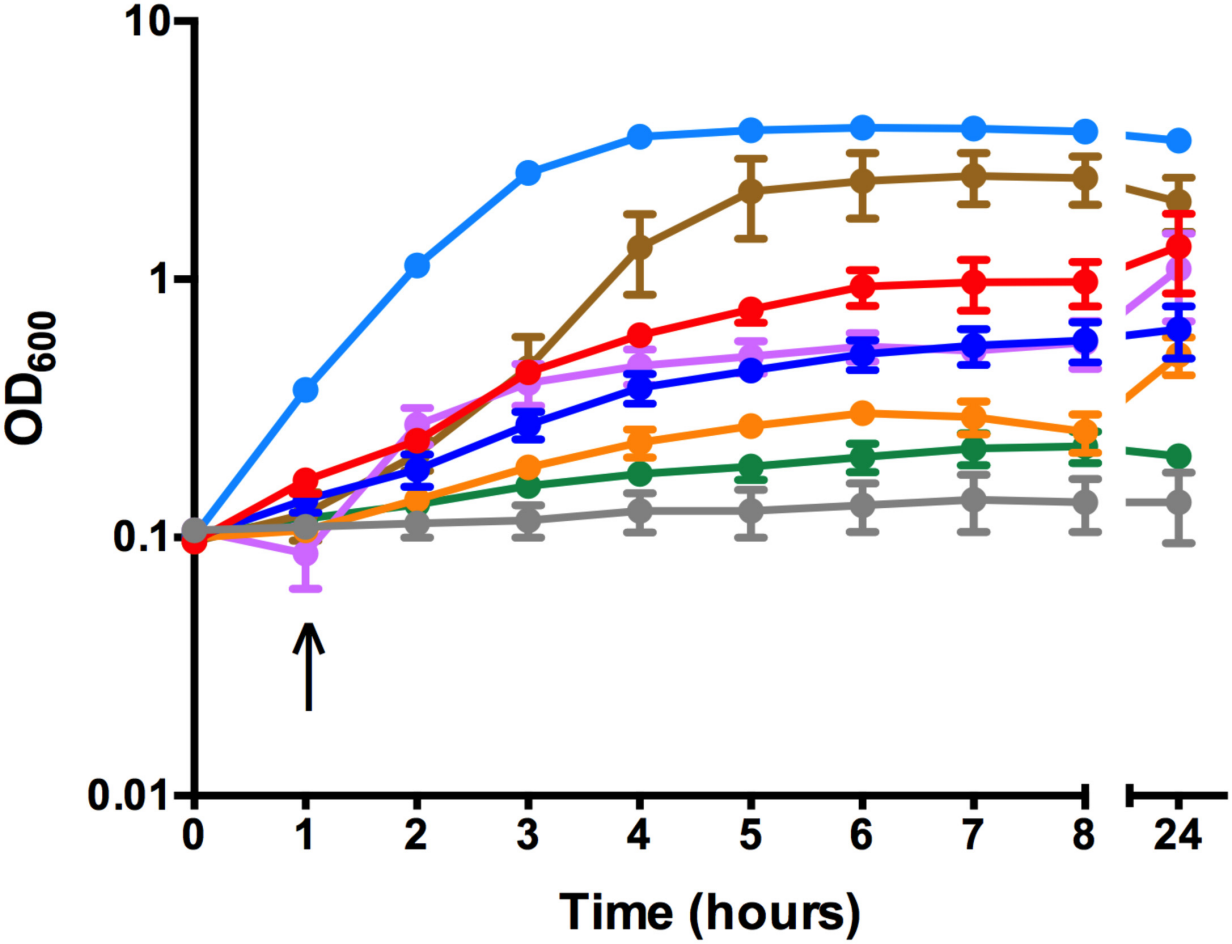
Growth curve of JIR325 using iron- or heme-containing proteins as the sole iron source. JIR325 was grown in pre-reduced tryptic soy broth under normal conditions (TSB) (light blue), iron-depleted conditions (TSB containing 150 μM 2,2-dipyridyl) (green) or iron-replete conditions supplemented with 200 μM FeCl_3_ (brown), 40 μM commercial Hb (predominantly metHb; purple), HbO_2_ at 25 μM (blue), HbO_2_ at 50 μM (red), HbCO at 50 μM (orange), or human holo-transferrin at 6.25 μM final concentration (grey). The arrow represents the time point at which the iron source was added. Bacterial growth was monitored by measuring the turbidity of culture samples at 600 nm (OD_600_). Error bars represent the standard error of the mean of n ≥ 3 independent experiments.

To determine the ability of *C*. *perfringens* to utilize various iron sources, strain JIR325 was grown *in vitro* in iron-depleted TSB media supplemented with host iron sources including human Hb and holo-transferrin, as well as an inorganic iron source, FeCl_3_ (iron-replete conditions). As controls, JIR325 was also grown in TSB alone (normal conditions) and in TSB containing 150 μM 2,2-dipyridyl, a chelator of free iron (iron-depleted conditions). The lack of growth of JIR325 in iron-depleted TSB indicated that iron was essential for its growth (Fig 1). The addition of 200 μM FeCl_3_ to the iron-depleted media restored growth to the level observed under normal conditions. Similarly, growth was restored in the presence of 40 μM commercial Hb (predominantly, metHb), 25 μM and 50 μM HbO_2_, albeit partially (Fig 1), but was not restored in 50 μM HbCO, under the conditions tested. Lower concentrations, including 10 μM of both the commercial Hb and freshly prepared HbO_2_ did not restore growth (data not shown). These findings indicated that JIR325 was capable of utilizing iron from FeCl_3_ and human Hb in both the metHb and HbO_2_ form. By contrast, the lack of growth of JIR325 in iron-replete media supplemented with human holo-transferrin (Fig 1) indicated that JIR325 was unable to utilize this host iron source for its *in vitro* growth under the conditions tested.

### Identification and transcriptional analysis of the *cht* locus

**Fig 2:**
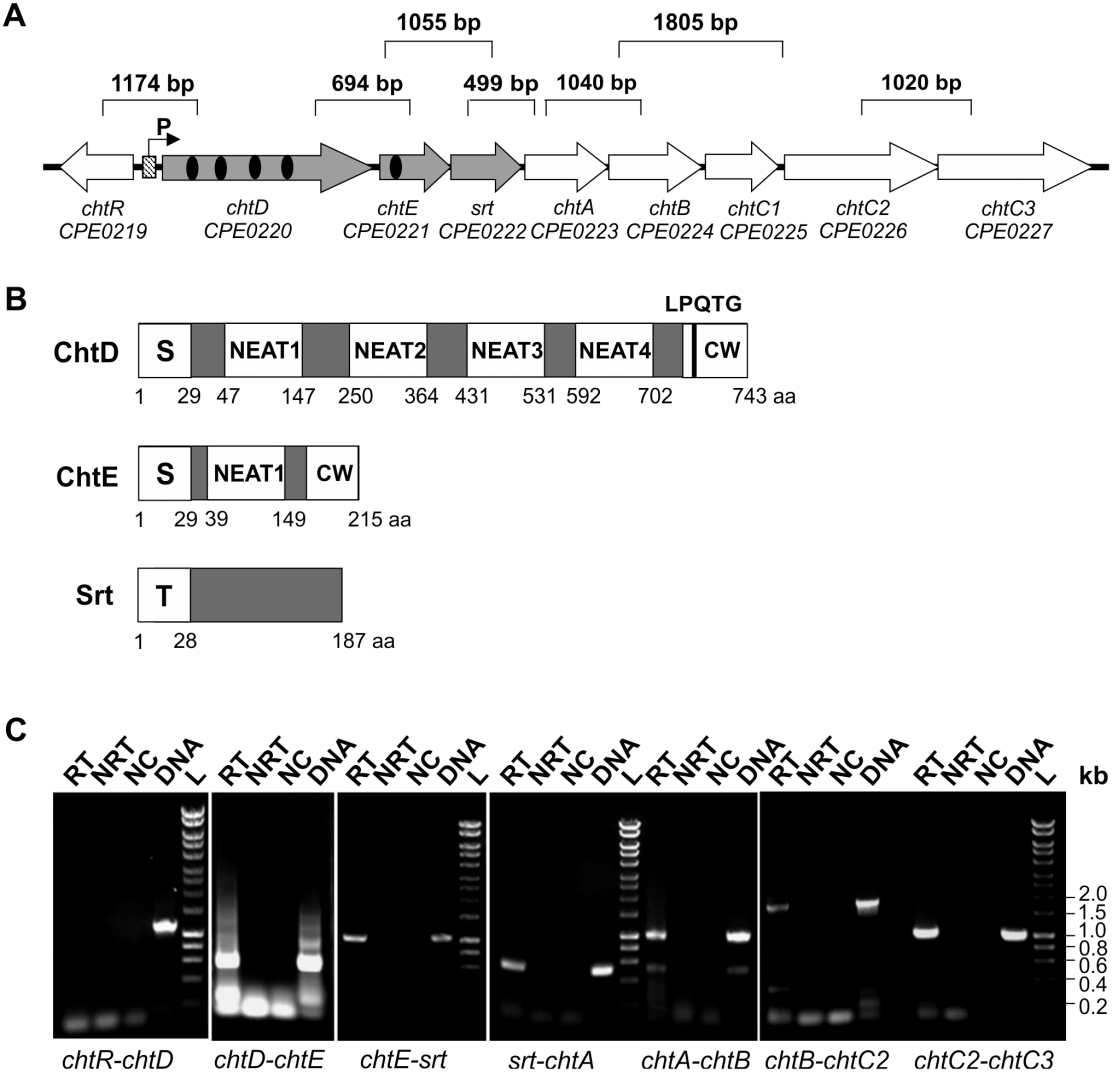
Characterization of the *cht* locus of JIR325. (A) Genetic organization of the *cht* locus in JIR325. The putative promoter (P) and the Fur box (diagonal tiled box) are located directly upstream of the *cht* locus. The NEAT domain(s) of ChtD and ChtE are shown as black oval(s). The product sizes for the gene junction analyzed by RT-PCR are indicated above the genes. (B) Schematic representation of the structural features of ChtD, ChtE and Srt. The signal peptide (S) is shown as is the cell wall sorting signal (CW) which contains a potential sortase recognition motif (LPQTG), potentially recognized and cleaved by the sortase transpeptidase to incorporate the protein into the bacterial cell envelope for surface display. The N-terminal transmembrane domain (T) of the sortase is also shown and is postulated to anchor the sortase protein to the cell membrane. (C) RT-PCR analysis of wild-type JIR325 cells grown to mid-logarithmic phase under iron-limited conditions (TSB containing 100 μM 2,2-dipyridyl). The isolated RNA was reverse transcribed into cDNA and PCR amplified using the appropriate primers. DNA standard sizes are indicated on the right of the gel. Lanes: RT- with reverse transcriptase (cDNA); NRT- no reverse transcriptase (RNA); NC- no template control (dH_2_O); DNA- JIR325 genomic DNA; L- Hyperladder 1kb (Bioline).

Studies in several Gram-positive bacteria have shown that heme uptake is mediated by Isd or Isd-like systems [7, 41, 42]. Therefore, the *cht* locus of JIR325 was investigated to determine its role in heme acquisition by *C*. *perfringens*. The *cht* locus of JIR325 comprises eight genes (Fig 2A), and encodes components similar to those of the Isd system from *S*. *aureus*. The *chtD* and *chtE* genes of JIR325 encode putative cell surface proteins containing four and one NEAT domain(s), respectively. These NEAT domains have amino acid sequence similarity to each other (22.0% to 44.9% identity) and to most NEAT domains of *S*. *aureus* (8.2% to 21.3%) and *B*. *anthracis* (12.6% to 31.4%) (Alignments shown in S1 Fig). ChtD has regions of similarity to the Isd hemoprotein receptors of *S*. *aureus*, which include an N-terminal signal peptide, a C-terminal cell wall sorting signal consisting of a LPQTG sortase recognition motif, a hydrophobic domain and a positively charged tail (Fig 2B). ChtE also has an N-terminal signal peptide and NKESS or SEATG residues at the C-terminus, potentially for sortase recognition. The Srt protein is a putative sortase (transpeptidase) (Fig 2A) belonging to the class D sortase family, as reported in a previous study [43, 44]. This protein contains an N-terminal transmembrane region (Fig 2B), suggesting that it may be localized to the cell membrane. Based on the prediction that ChtD and ChtE are cell surface proteins, it was postulated that ChtD and/or ChtE were exported through the general secretory pathway and tethered to the *C*. *perfringens* cell surface by the Srt enzyme.

Downstream of *srt*, three genes designated as *chtA*, *chtB* and *chtC1*, are predicted to encode a periplasmic binding protein, a permease and an ATP-binding protein of a putative ABC transport complex, respectively (Fig 2A). Two other ORFs downstream of *chtC1*, known as *chtC2* and *chtC3*, encode putative permease/ATPase fusion proteins containing a transmembrane domain and an ATPase domain at the N- and C-terminal regions, respectively.

The genes in the *cht* locus are organized in an operon-like structure, with several genes either containing short intergenic regions or overlapping one another, suggesting that they are co-transcribed. RT-PCR analysis was performed across the intergenic junctions from *chtD* to *chtC3*, including the adjacent genes and the downstream gene (as negative controls). The absence of a transcript for the *chtR* and *chtD* junction indicated that these genes were not co-transcribed, as expected since these genes are divergently orientated (Fig 2C). RT-PCR of the gene junctions from *chtD* to *chtC3* produced gene transcripts of the expected sizes (Fig 2C), whereas no product was observed for the gene junction between *chtC3* and the gene immediately downstream (data not shown). These results indicate that the *cht* locus of JIR325 consisted of eight genes, from *chtD* to *chtC3*, and that these genes were co-transcribed.

Sequence analysis of the *cht* locus promoter region also indicated the presence of a putative Fur binding site, or Fur box (5’-CATTATTGATAATCATTTTCA-3’), located 33 bp upstream of the *chtD* start codon (Fig 2A). In many bacterial species, Fur acts as a transcriptional repressor of iron acquisition genes by controlling the promoter activity of a gene in response to iron availability [45, 46].

### ChtD, ChtE and Srt are expressed under iron-limiting conditions

**Fig 3:**
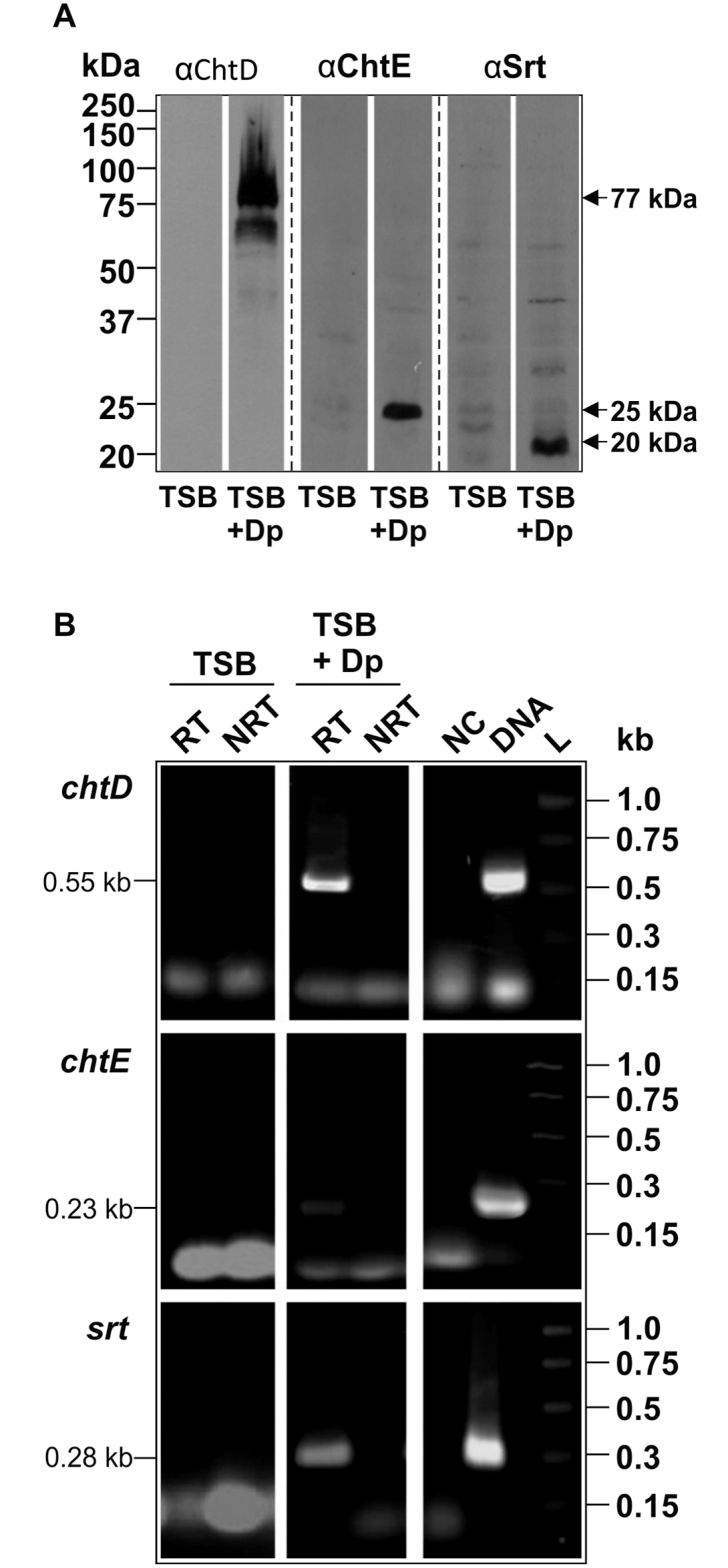
Expression of ChtD, ChtE and Srt is upregulated under iron-limited conditions. (A) Western blot of whole cell lysates of JIR325 grown under normal conditions (TSB) or under iron-limited conditions (TSB containing 100 μM 2,2-dipyridyl, TSB + Dp). The cell lysates were probed with polyclonal antibodies to ChtD (anti-ChtD), ChtE (anti-ChtE) and Srt (anti-Srt). Bound antibodies were detected with HRP-conjugated goat anti-rabbit IgG (Millipore). The protein marker sizes (in kDa) are indicated on the left of the blot. (B) RT-PCR of *chtD*, *chtE* and *srt* on RNA isolated from JIR325 grown to mid-logarithmic phase under normal (TSB) and iron-depleted (TSB + 150 μM 2,2-dipyridyl, TSB + Dp) conditions. The *chtD* (576 bp), *chtE* (230 bp) and *srt* (276 bp) gene regions were PCR amplified using the appropriate primer pairs (S1 Table). DNA standard sizes (kb) are stated on the right of the gel. Lanes: RT- with reverse transcriptase (cDNA); NRT- no reverse transcriptase (RNA); NC- no template control (dH_2_0); DNA- JIR325 genomic DNA; L-PCR markers (Promega).

The presence of a putative Fur box in the *isd* promoter region suggested that these genes may be regulated in an iron-dependent manner. Therefore, the production of proteins encoded by the *cht* locus was examined during growth under normal and iron-limited conditions in TSB. Western blot analysis, using specific polyclonal antibodies raised against ChtD, ChtE and Srt, detected immunoreactive bands approximately 77 kDa, 25 kDa and 20 kDa in size, corresponding to ChtD, ChtE and Srt, respectively. These bands were detected in cell lysates of JIR325 grown under iron-limited conditions (TSB + 100 μM 2,2-dipyridyl), but not from cells grown under normal conditions (TSB) (Fig 3A). These findings provide evidence that the *cht* locus of JIR325 is up-regulated under iron-limited conditions. RT-PCR analysis of RNA isolated from JIR325 cells grown under similar conditions confirmed that the *chtD*, *chtE* and *srt* genes were transcribed under iron-limited conditions, but not under normal growth conditions (Fig 3B). These results indicate that the *cht* locus of JIR325 is up-regulated under iron-limiting conditions and that this iron-dependent regulation occurs at the transcriptional level.

### ChtD and ChtE bind heme

**Fig 4:**
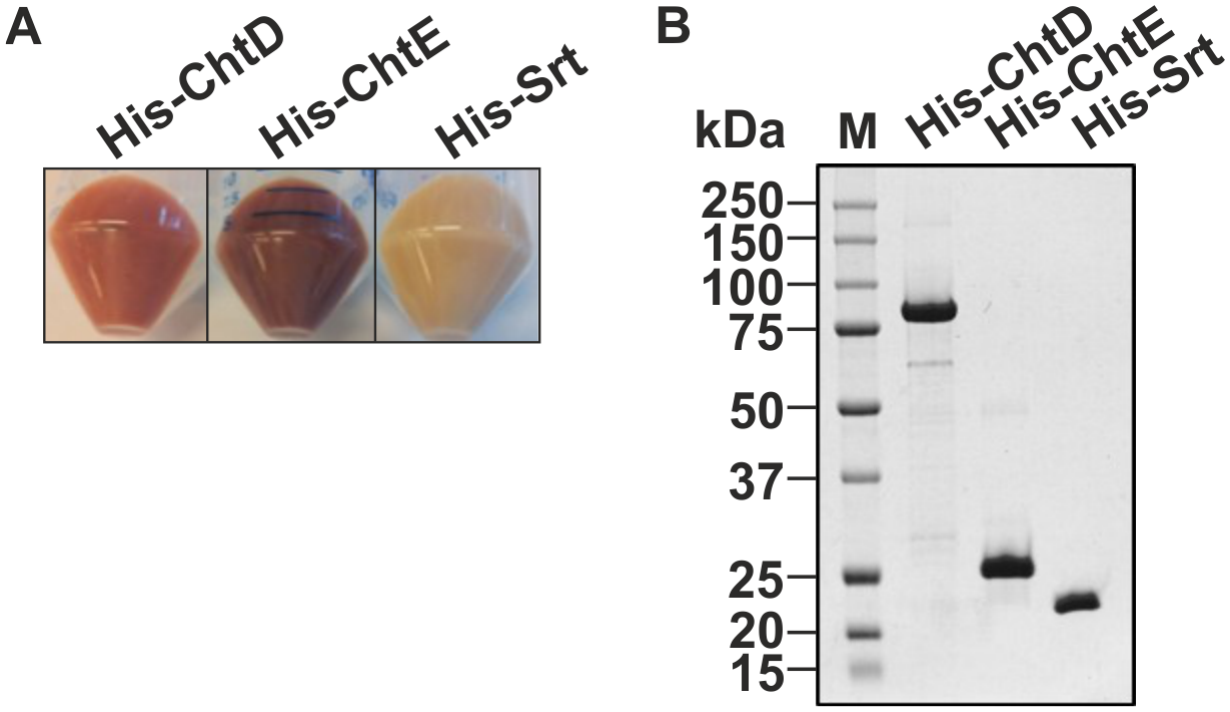
ChtD, ChtE and Srt recombinant proteins were expressed and purified. (A) The cell pellets of *E*. *coli* overexpressing the His-ChtD, His-ChtE and His-Srt recombinant proteins. The red colored cell pellets containing His-ChtD and His-ChtE indicate the presence of heme. (B) Purified His-ChtD (77 kDa), His-ChtE (25 kDa) and His-Srt (20 kDa) proteins were analyzed on a Coomassie-stained 10% SDS-PAGE gel. The band sizes are indicated on the right and the protein marker (M) sizes are shown on the left of the gel.

To determine the role of the JIR325 *cht* locus in heme acquisition, the ability of the NEAT proteins ChtD and ChtE to bind heme was determined. The Srt protein was used as a negative control as it was not predicted to bind heme. The genes encoding ChtD (residues 31 to 711), ChtE (residues 31 to 191) or Srt (residues 29 to 187) were cloned into a pET28a(+) expression vector to generate N-terminal His_6_-tagged fusion proteins. The cloned region included the DNA sequence that encoded the NEAT domains (for ChtD and ChtE), but not the N-terminal signal peptide and/or transmembrane domain, to obtain soluble proteins. The cell pellets of *E*. *coli* BL21(DE3) expressing His-ChtD or His-ChtE were red in color, unlike the pellets containing the His-Srt protein, which were cream in color (Fig 4A). These findings suggest that His-ChtD and His-ChtE were able to sequester heme during their expression in *E*. *coli*, as reported in several studies [47, 48]. Purification of the His-tagged fusion proteins by affinity chromatography and SDS-PAGE showed the expected sizes for His-ChtD, His-ChtE and the control protein His-Srt, at 77 kDa, 25 kDa and 20 kDa, respectively (Fig 4B).

**Fig 5:**
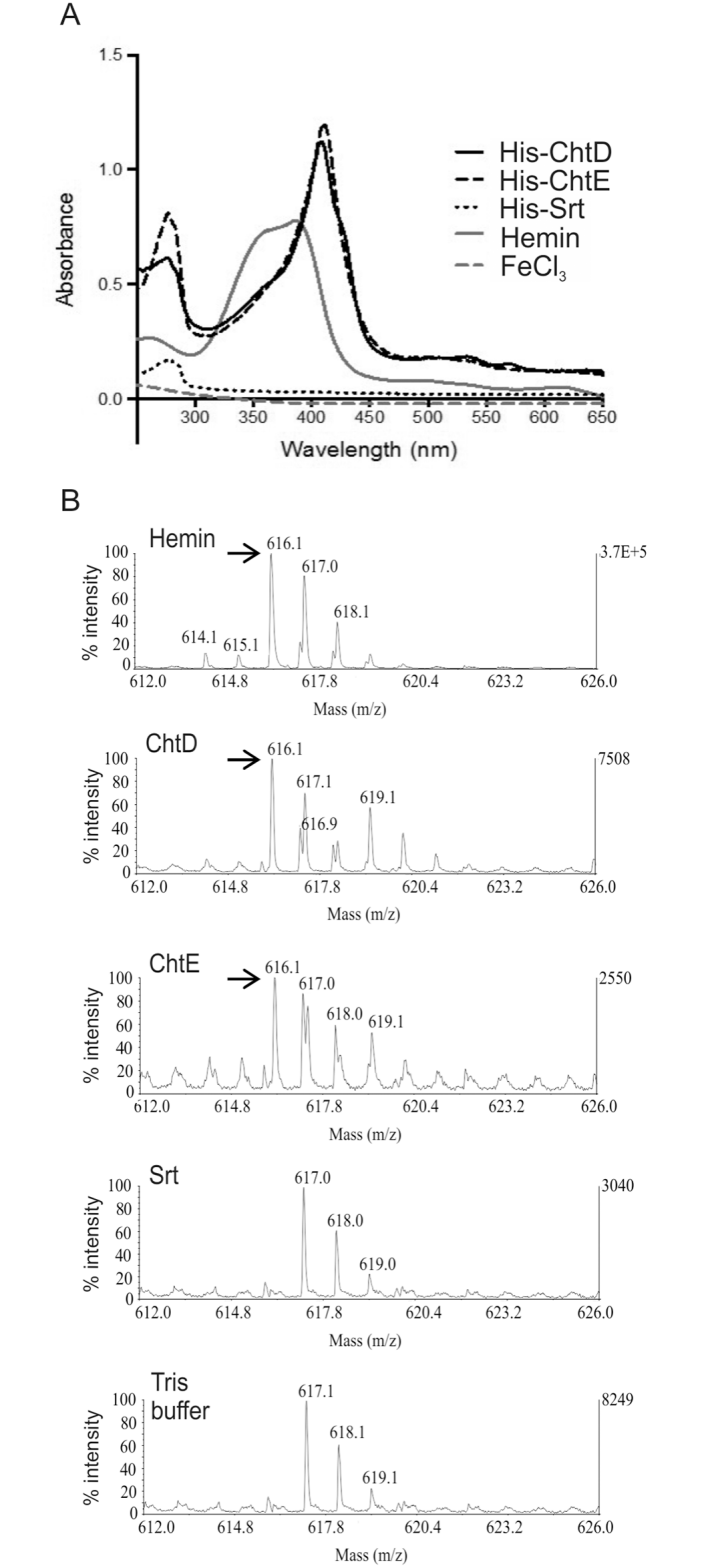
ChtD and ChtE recombinant proteins bind heme. (A) Each purified recombinant protein and a hemin only sample was analyzed by absorbance spectroscopy between wavelengths of 250 to 650 nm. The presence of heme bound to the proteins was determined by a Soret absorbance at ~400 nm. (B) Detection of heme by LC-MS analysis of each purified recombinant protein, a hemin only sample and Tris buffer as a control. A single charged heme species, indicated by the arrow (→), is predicted to have a mass of 616 kDa.

To assay for bound heme, the absorbance spectrum from 250 to 650 nm was analyzed for each recombinant protein. An absorbance peak at ~400 nm (Soret band) is a characteristic feature that is indicative of heme bound to protein [48, 49]. The absorbance spectra of the His-ChtD and His-ChtE proteins revealed a Soret band at ~400 nm (Fig 5A), which indicated that His-ChtD and His-ChtE had co-purified with heme. In contrast, a hemin only preparation produced a broad absorbance maximum between 350 to 400 nm [49]. The His-Srt protein, which was not expected to bind heme, did not have a Soret peak at ~400 nm. These observations also indicated that the ability of His-ChtD and His-ChtE to bind heme was not due to the non-specific binding of the His_6_-tag to heme.

To confirm the co-purification of endogenous heme with His-ChtD and His-ChtE, the purified proteins were subjected to LC-MS analysis. The Tris buffer used for the purification of the recombinant proteins, and a hemin preparation, were also analyzed as negative and positive controls, respectively. Samples containing the heme species were predicted to produce a peak at 616 m/z, which is representative of a single charged heme molecule [50]. LC-MS analysis of His-ChtD and His-ChtE samples revealed a peak at 616 m/z, which was consistent with that observed for hemin (Fig 5B). These results indicate that heme was present in the His-ChtD and His-ChtE preparations. The His-Srt sample did not produce an equivalent peak, which again was consistent with the protein not containing bound heme. Additionally, the absence of a 616 m/z peak in the protein buffer alone indicated that the 616 m/z peak was only associated with a heme molecule. A 617.0 m/z peak was observed in all of the samples including the protein buffer, which did not contain heme, suggesting that this peak is non-specific and is not indicative of heme. Taken together, these observations provide evidence that ChtD and ChtE are able to sequester heme.

### *chtDE* and *srt* mutants are not defective in hemoglobin and ferric chloride utilization

To examine the functional role of the *C*. *perfringens* JIR325 *cht* locus in heme acquisition, allelic exchange was used to construct an *chtDE* deletion mutant (JIR12755) by replacing the *chtD* and *chtE* genes with a chloramphenicol resistance cassette, *cat*(P). In addition, an *srt* mutant (JIR12614) was constructed by insertional inactivation using Targetron technology, which introduced a group II intron with an *erm*(B) erythromycin resistance gene into the *srt* gene. PCR analysis and Southern hybridization confirmed the deletion of the *chtDE* genes and the insertional inactivation of the *srt* gene in the respective mutants (data not shown).

**Fig 6:**
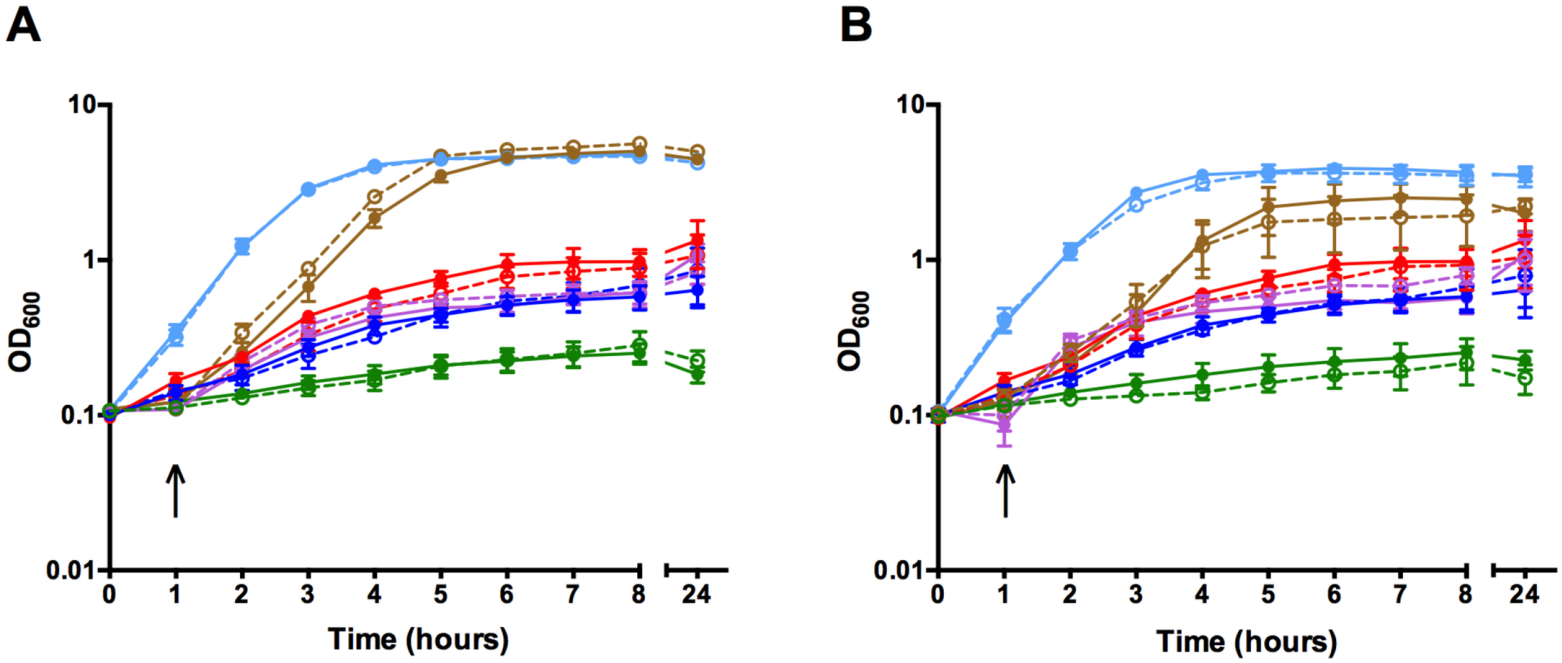
The cht locus is not required for efficient iron and heme utilization. Growth curve of the wild type (closed symbol, solid line) and the (A) *chtDE* mutant (open symbol, dotted line) or (B) *srt* mutant (open symbol, dotted line) under normal conditions (light blue), iron-depleted conditions (green) and iron-replete conditions with 200 μM FeCl_3_ (brown), 40 μM commercial Hb (predominantly metHb, purple) or HbO_2_ at 25 μM (blue) or 50 μM (red) as the sole iron source. The arrow represents the time point at which the iron source was added. Bacterial growth was monitored by measuring the turbidity of the culture at 600 nm (OD_600_). Growth of the wild type is shown with the solid lines and the mutant strains as the dotted lines. Error bars represent the standard error of the mean of n≥ 3 independent experiments.

To determine whether the *cht* locus was involved in the acquisition of iron from Hb, the *in vitro* growth of the wild-type and the isogenic *chtDE* and *srt* mutants was compared under normal, iron-depleted and iron-replete conditions containing human Hb (metHb or HbO_2_) or FeCl_3_ as the sole iron source. The growth of the *chtDE* and *srt* mutants was similar to the wild-type strain under normal conditions (Fig 6), while no growth was observed for all strains grown under iron-depleted conditions, as previously observed for the wild-type strain (Fig 1). Under iron-replete conditions, the *chtDE* (Fig 6A) and *srt* (Fig 6B) mutants were still able to utilize FeCl_3_ or human Hb (metHb or HbO_2_) at wild-type levels. This observation indicated that under the conditions tested, the *cht* locus was not essential for Hb or FeCl_3_ utilization in JIR325.

### The inactivation of *srt* does not affect the cell wall-anchoring of ChtD and ChtE

**Fig 7:**
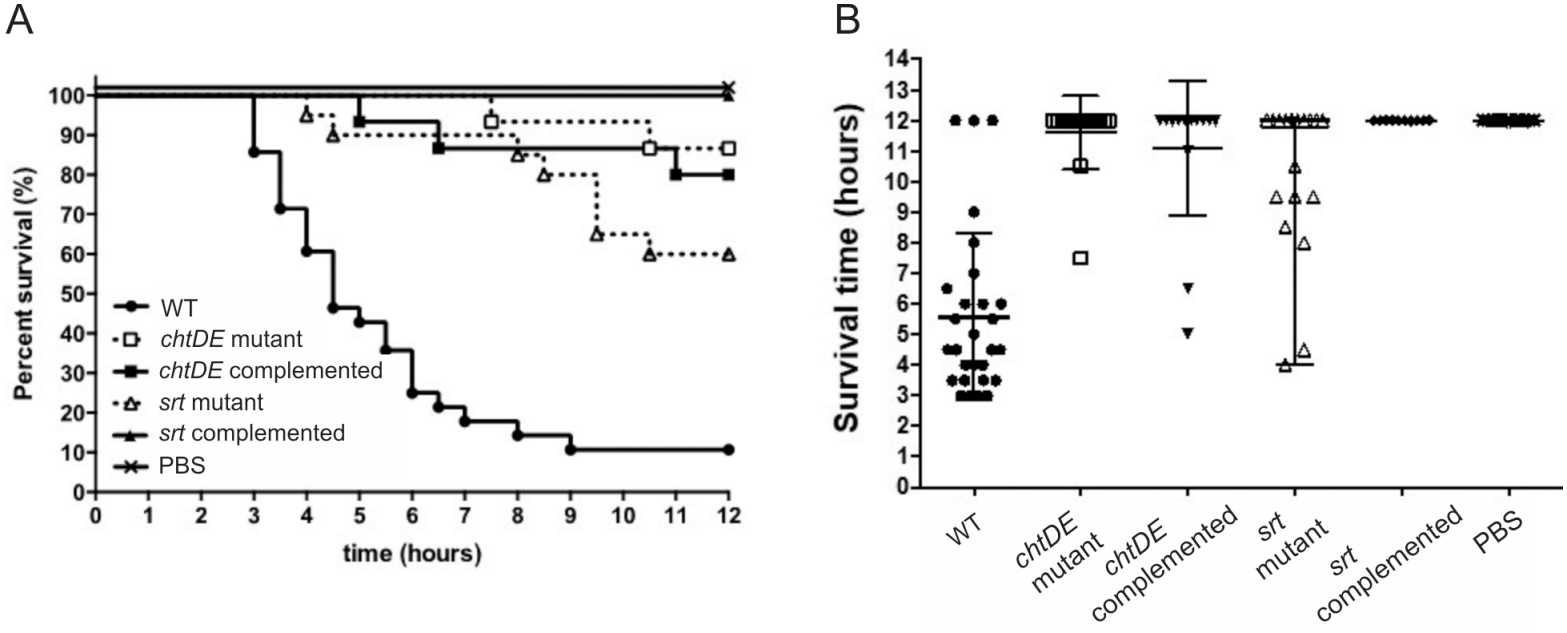
ChtD, ChtE and Srt proteins are localized to the cell envelope of *C*. *perfringens*. The whole cell lysate (W) or the supernatant (S) and cell pellet (P) fractions containing the cytoplasmic and cell envelope proteins, respectively, were isolated from the wild type and *srt* mutant. The cell fractions were analyzed by Western blotting with polyclonal antibodies to ChtD (anti-ChtD), ChtE (anti-ChtE) or Srt (anti-Srt). The protein marker sizes (in kDa) are indicated on the left of the blot.

The cellular localization of ChtD, ChtE and Srt was examined by separating the whole cell lysates of JIR325 into cytoplasmic and cell envelope fractions. Western blot analysis using polyclonal antibodies to ChtD, ChtE or Srt, indicated the presence of ChtD in both the cell envelope and cytoplasmic fractions (Fig 7). In contrast, the ChtE and Srt proteins were associated only with the cell envelope fraction. The presence of ChtD, ChtE and Srt in the cell envelope fraction was consistent with the predicted role of ChtD and ChtE as cell surface proteins, and the role of the sortase as a membrane-anchored enzyme.

To determine whether Srt was involved in the anchoring of ChtD and ChtE to the cell wall, the cellular localization of these proteins was analyzed in the *srt* mutant as before. The results showed that the localization of both ChtD and ChtE was unaltered in the *srt* mutant (Fig 7). Immunoreactive species were not observed in any of the cellular fractions from the *srt* mutant when they were probed with the Srt polyclonal antibody, confirming that Srt was not produced in the mutant strain. These results suggested that this Srt protein was not required to localize ChtD and ChtE to the cell wall of *C*. *perfringens* JIR325, under the conditions tested.

### Virulence studies of the isogenic *chtDE* and *srt* mutants in a mouse myonecrosis model

**Fig 8:**
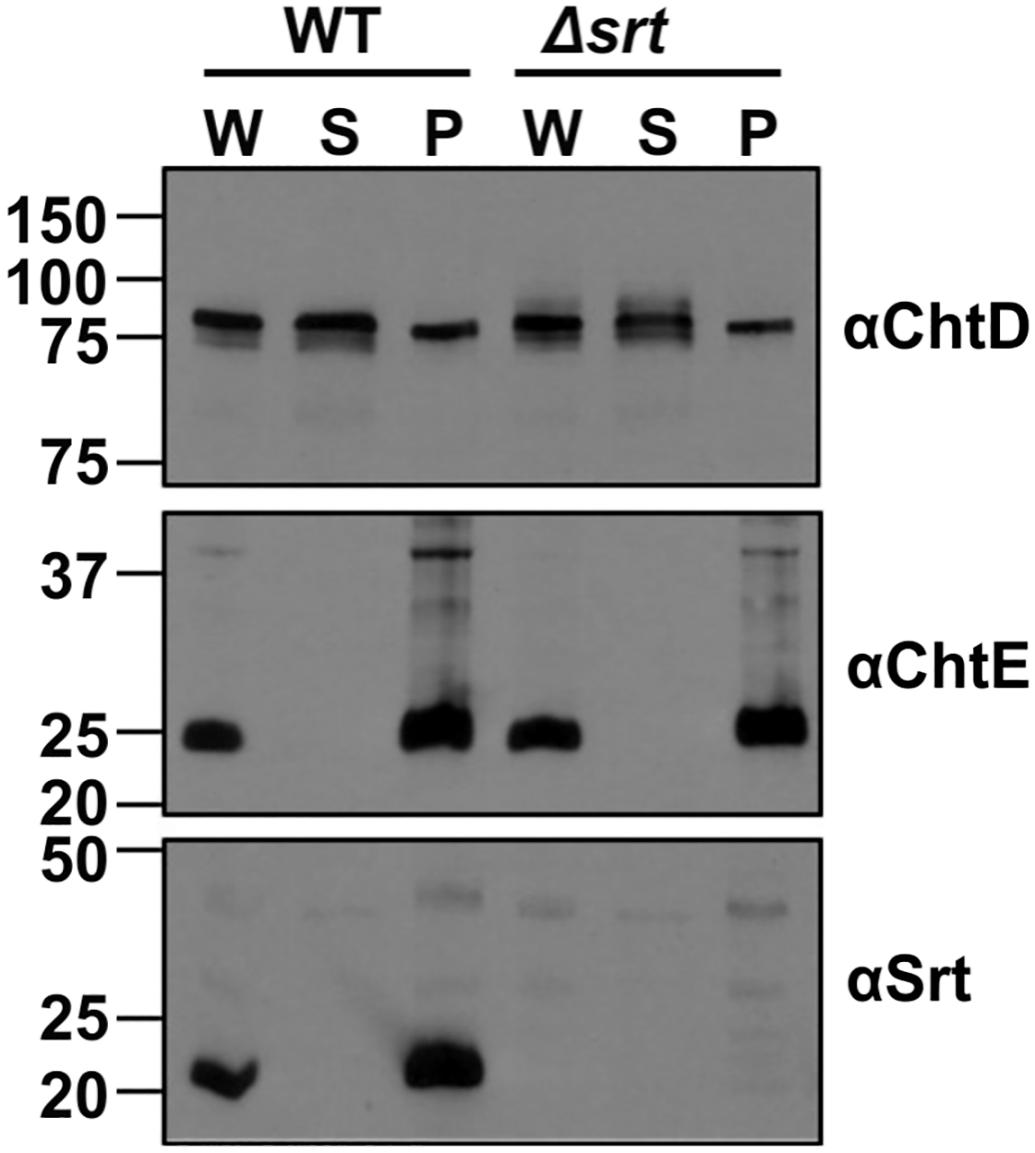
Virulence of *chtDE* and *srt* isogenic strains in a mouse myonecrosis model. BALB/c mice were infected with the wild type (n = 28), *chtDE* mutant (n = 15), *chtDE in cis* complemented (n = 15), *srt* mutant (n = 20) and *srt* complemented strains (n = 10). Disease progression of the mice was monitored for 12 h and the (A) survival curve was recorded. (B) Survival results shown as survival time, with the error bars representing the SEM. p<0.0001, log rank Mantel-Cox test for survival curve (A) and Mann Whitney U test for survival time (B) comparing wild type with mutant and complemented strains.

To investigate the contribution of the *cht* locus to virulence, cells of either the wild-type strain or the isogenic mutants were injected into the right thigh muscle of BALB/c mice, using our established mouse myonecrosis model [40]. Mice infected with the *chtDE* or *srt* mutants showed reduced disease in most of the disease parameters tested (data not shown) and had an increased mean survival time (*chtDE* mutant, 11.6 ± 0.3 h; *srt* mutant 10.4 ± 0.6 h) compared to those infected with the wild-type strain, which progressively succumbed to disease from 2 to 3 h post-infection (mean survival time, 5.6 ± 0.5 h) (Mann Whitney U test, p < 0.0001) (Fig 8A and 8B). In an attempt to validate these results, the *chtDE* and *srt* mutants were complemented *in cis* and the isogenic complemented strains were virulence tested using the same model. The *chtDE* and *srt* complemented strains (JIR12773 and JIR12861, respectively) were however, also attenuated for virulence (p < 0.0001). Viable cell counts were performed pre- and post-infection and showed that the bacterial numbers recovered from mice infected with the mutants were comparable to those infected with the wild-type strain. These results suggested that the *chtDE* and *srt* mutations were not responsible for the reduced virulence observed with the mutants.

### Whole genome sequencing of the *chtDE* and *srt* mutants

To determine if the inability to complement the *chtDE* and *srt* mutants was due to spontaneous secondary mutations, whole genome sequencing of the wild-type, *chtDE*, and *srt* strains was undertaken. Among the mutations identified in the *in cis* complemented *chtDE* mutant was a single nucleotide polymorphism (SNP) in *feoA*, which resulted in an A44D amino acid substitution (Table 2). The *feoA* gene is upstream of *feoB*, which in other organisms, encodes a ferrous iron transporter and is involved in bacterial virulence [51, 52]. Three mutations that were common to the *chtDE* and *srt* mutants were also identified (Table 2). These mutations resulted in the deletion of amino acid A163 in a putative ferrodoxin hydrogenase (CPE0276), a frameshift in a hypothetical protein (CPE2000) and a D443N substitution in a putative BaeS sensor histidine kinase (Table 2). The impact of these mutations on protein function, as well as in combination with the other mutations in the *chtDE* isogenic strains and the *srt* mutant remains to be determined.

**Table 2:** Distribution of SNPs in the *chtDE* and *srt* strains.

Nucleotide change (comparison to wild-type sequence			Genome position	Amino acid change	Putative protein function
Δ*chtDE*	Δ*chtDE* (KI)	*srtΩermB*			
CTG deletion	CTG deletion	CTG deletion	356016–356018	A163-	Ferrodoxin hydrogenase (CPE0276)
C → A	C → A		652650	P10Q	ABC transporter (CPE0526)
C → A	C → A		714375	L371I	Two component sensor histidine kinase YesM (CPE0574)
C → A	C → A		850962	A102D	Amino phosphoribosyltransferase (CPE0683)
	C → A		1051900	F205L	NADH-dependent butanol dehydrogenase or iron-containing alcohol dehydrogenase (CPE0858)
	G → T		1169378	E602D	Hypothetical protein (CPE0969)
	G → T		1339585	E134D	Triphosphoribosyl-dephospho-CoA synthase, CitG (CPE1145)
		G → T	1381825	M1I	Phosphofructokinase (CPE1185)
	C → A		1931454	A44D	FeoA (CPE1659)
- → C	- → C	- → C	Insertion after 2292378	Frameshift	Hypothetical protein (CPE2000)
	C → A		2323940	A559D	DnaK molecular chaperone (CPE2033)
G → T	G → T		2644353	A54S	Electron transfer flavoprotein beta-subunit (CPE2299)
		G → T	2716228	E405*	Phosphoenolpyruvate-protein phosphotransferase (CPE2357)
G → A	G → A	G → A	2847860	D443N	Two component sensor histidine kinase, BaeS (CPE2487)

* stop codon, KI- complemented *in cis*, *ΩermB-* insertional inactivation using the *erm*(B) gene

## Discussion

Most bacteria have developed diverse mechanisms to acquire iron from the host since this element is critical for bacterial growth and the subsequent development of an infection [4, 14]. In this study, we showed that the *in vitro* growth of *C*. *perfringens* strain JIR325 was supported in media containing commercially sourced human Hb (predominantly metHb), freshly prepared HbO_2_ or FeCl_3_ as the sole iron source (Fig 1). The ability of *C*. *perfringens* to utilize Hb, albeit at high concentrations, is in agreement with the literature on numerous bacteria, including human pathogens such as *S*. *aureus*, *B*. *anthracis* and *N*. *meningitidis*, as Hb is the major reservoir of iron in the mammalian host [7, 15, 53]. *C*. *perfringens* type A produces cytotoxins such as α-toxin and perfringolysin O, which are able to lyse red blood cells [25]. The production of cytolytic toxins and/or hemolysins is suggested to contribute to the release of Hb and the subsequent acquisition of heme by pathogenic bacteria [54, 55].

The use of freshly prepared human Hb in this study was important since lyophilized human Hb is typically oxidized [56] and can contain denatured protein and free heme, making it unsuitable as a sole iron source, to test for microbial growth [57]. Growth on freshly prepared HbO_2_ could be attributed to uptake of Fe(II) or Fe(III) heme. Thus, whilst dissociation of O_2_ typically regenerates Fe(II) heme, oxidation to Fe(III) heme is favored under low O_2_ conditions [58]. In contrast, CO binds Hb ~200 times more strongly than O_2_ [59] conferring protection against heme oxidation and dissociation from Hb [60]. Failure of HbCO to support the growth of *C*. *perfringens* could be due to lower rates of heme dissociation from Hb, and/or the absence of hemophores required to utilize this form of Fe(II) heme. Notably, most hemophores show a strong preference for binding to Fe(III) over Fe(II) heme [61, 62]. The ability to utilize specific forms of Hb has previously been noted. For example, a NEAT domain of the primary Hb receptor from *S*. *aureus*, IsdB, was shown to be capable of extracting heme from metHb but not from HbCO [63].

The utilization of FeCl_3_ by *C*. *perfringens* is consistent with the presence of four putative siderophore-mediated iron uptake systems, which in many pathogenic bacteria, are used to acquire ferric iron from host compounds such as ferritin, transferrin or lactoferrin [64–66]. In contrast, the inability of human holo-transferrin to support the *in vitro* growth of *C*. *perfringens* could be due to a lack of receptors or siderophores for transferrin, as reported for *S*. *pyogenes* and *S*. *pneumoniae* [67, 68]. Importantly, the growth of JIR325 under iron-replete conditions indicated that the wild-type strain was unable to grow under iron-depleted conditions due to the restricted availability of iron and not because the iron chelator 2,2-dipyridyl used in the growth assay was cytotoxic to the *C*. *perfringens* cells.

The presence of multiple mechanisms for heme/iron uptake by *C*. *perfringens* suggests that it may use these systems to adapt to changes in both the availability and type of iron sources within the host, as observed for *P*. *aeruginosa* and *S*. *aureus* [42, 69]. High concentrations of Hb (25 μM and 50 μM HbO_2_) were required to effectively support the growth of *C*. *perfringens* strain, JIR325, in contrast to that reported for *S*. *aureus* (10 nM HbO_2_) [70], suggesting that Hb may not be the preferred iron source for the *in vitro* growth of this organism, under the conditions tested. Furthermore, recent studies have shown that FeoB from JIR325 appears to be the major protein required for iron acquisition in this strain, since a *feoB* mutant had an approximately 99% reduction in total iron content compared to the wild-type strain [27].

The genes encoding two NEAT proteins, a putative sortase and a putative membrane transport system were shown to be co-transcribed in JIR325. Similar genes in the Isd system of *S*. *aureus* and *B*. *anthracis* are also encoded in an operon, with the smaller *B*. *anthracis isd* operon predicted to encode the cell surface NEAT proteins [7]. Additionally, three of the cell surface proteins (IsdA, IsdB and IsdH) of *S*. *aureus* and the heme oxygenases of *S*. *aureus* and *B*. *anthracis* are encoded as single transcriptional units [22, 71]. In *C*. *perfringens*, a *hemO* gene, located five ORFs upstream of *chtD*, has been shown to encode a heme oxygenase that mediates the degradation of heme to biliverin and iron in strain 13, the parent of JIR325 [72]. These findings suggest that the *C*. *perfringens cht* locus encodes a complete heme acquisition system, although the mechanism of heme uptake may differ to that of the well-characterized Isd system of *S*. *aureus*.

Transcriptional and Western blot analyses revealed that when JIR325 was grown under iron-limited conditions there was an increase in both the transcription of the *chtD*, *chtE* and *srt* genes, and the production of the respective proteins (Fig 3). These results provide evidence that the expression of the *cht* locus is regulated by the availability of iron and are consistent with the presence of a putative Fur box in the *chtD* promoter region of the locus, as has been observed in the Isd systems of *S*. *aureus* and *L*. *monocytogenes* [4, 6]. The expression of iron uptake genes in many bacteria is regulated by the levels of extracellular iron present in the environment and is usually repressed, in the presence of excess iron, by the binding of a Fur protein to Fur boxes in the promoter region of regulated genes [8, 45]. Whilst three genes have been annotated as potential Fur homologues in strain 13 [26], it is not known which of these genes encode a functional Fur protein

Absorbance spectroscopy and LC-MS analysis indicated that the NEAT proteins, ChtD and ChtE, were able to bind Fe(III) heme. The mechanism of heme binding by these proteins is not known, but studies on the *S*. *aureus* and *B*. *anthracis* hemoprotein receptors have shown that individual NEAT domains mediate heme binding [49, 73, 74], which suggests that the NEAT domains of ChtD and ChtE have a similar functional role. Structural and biochemical studies on the NEAT domains of IsdA from *S*. *aureus* and IsdX2 from *B*. *anthracis* indicate that heme is coordinated by an invariant tyrosine residue located on the β7 strand of the protein [11, 46]. A second invariant tyrosine side chain is located four residues away in the same β strand, and is hydrogen bonded to the coordinating tyrosine side chain. These tyrosine residues are conserved in the NEAT domains of many reported heme-binding proteins [13, 74, 75]. However, the ChtD and ChtE NEAT domains studied here lack these invariant tyrosine residues (S1 Fig), suggesting that heme coordination in these proteins may occur by a different mechanism. The Shp heme binding protein of *S*. *pyogenes* has a unique heme binding mechanism involving two methionine residues (Met-66 and Met-153), although the protein is folded into an immunoglobulin-like β-sandwich fold similar to other NEAT domains [76]. Heme is transferred from Shp to the lipoprotein component of the heme transporter HtsA in this organism, a process facilitated through the displacement of the Met-66 and Met-153 residues of Shp by the Met-79 and His-229 residues of HtsA, respectively [77].

*C*. *perfringens* may utilize a similar heme acquisition pathway since the NEAT domain of its ChtE protein has 25% identity to Shp and the putative periplasmic binding protein, ChtA, has 32% identity to HtsA from *S*. *pyogenes*. The equivalent Met or His residues in Shp and HtsA are also conserved in ChtE and ChtA (S1 Fig). Additionally, the last NEAT domain of ChtD has 27% identity to NEAT5 of IsdX2 in *B*. *anthracis*, which is known to be essential for scavenging heme from metHb [78]. In IsdX2, this process requires an amino acid with an amine side chain within the 3_10_ helix of the NEAT5 domain [78]. The presence of an arginine in ChtD at a similar position is consistent with a similar mode of interaction between ChtD and Hb, however, further studies are required to examine this hypothesis. In addition to heme binding, individual NEAT domains can also mediate the extraction of heme from Hb and the transfer of heme between NEAT domains [49]. Therefore, the presence of multiple NEAT domains in ChtD suggests that it may be able to perform multiple roles in heme acquisition, potentially to increase the efficiency of heme uptake.

The ChtD, ChtE and Srt proteins were localized to the cell envelope of JIR325, where ChtD and ChtE were found to contain potential sorting motifs (LPQTG and NKESS, respectively) at their C-terminus. This association coupled with their heme binding ability, suggests that these proteins act as receptors for hemoprotein binding and may be processed for cell wall anchoring by the membrane-anchored Srt protein. A recent biochemical study of the Srt protein of *C*. *perfringens* strain 13 indicated that it preferentially recognised the LPQTG motif [44]. Additionally, in *S*. *aureus*, *B*. *anthracis* and *L*. *monocytogenes*, the *isd* locus encodes a sortase B protein, which is iron-regulated and anchors a subset of cell surface proteins bearing the NPQTN, NPKTH and NAKTN (or NPKSS) sorting motifs, respectively [14, 22, 79, 80]. Mutation of the *srtB* gene in *L*. *monocytogenes* resulted in the absence of a hemin binding protein, SvpA, in the peptidoglycan layer, however, a lower molecular weight SvpA species was observed in the supernatant [81]. In our system, the inactivation of *srt* did not affect the cell envelope localization of ChtD and ChtE in JIR325. The efficient activity of Srt has been suggested to be dependent on temperature and may require a substrate-induced conformational change for binding to LPQTG motifs [44]. Although similar conditions were used to assess the activity of the *C*. *perfringens* Srt protein, a lack of phenotype in the *srt* mutant suggests that ChtD may require different conditions in order to be properly anchored to the cell wall. Alternatively, ChtD and/or ChtE may be anchored to the cell wall by other putative sortases encoded by JIR325, or tethered to the cell wall by their C-terminal hydrophobic regions and short charged tails as suggested for the cell surface heme binding proteins Shp and Shr of *S*. *pyogenes* and HtaA of *Corynebacterium diphtheriae* [9, 48, 82]. Therefore, the functional role of the sortase encoded by the *cht* locus of JIR325 remains unknown.

Several studies have reported that the Isd system is required for the virulence of *S*. *aureus* [15, 16], although this result may be dependent on the specific conditions used in the animal infection model [83]. Studies in a mouse myonecrosis model showed that the *C*. *perfringens chtDE* and *srt* mutants were attenuated for virulence, although the virulence defects were not restored by complementation with the respective wild-type genes (Fig 7). The reduced virulence of these mutants may be the result of the specific mutation in the Cht system, the presence of SNPs in potential virulence-associated genes, as detected by genomic sequence analysis, or a combination of these mutations. SNPs were detected in genes encoding proteins such as FeoA, an ABC transporter and/or a phosphoenolpyruvate-phosphotransferase protein, all of which have been shown to be involved in the virulence of other bacteria [70, 84–88]. These secondary mutations had no effect on the growth of the *chtDE* or *srt* mutants in the presence of human Hb or FeCl_3_, but they appear to have contributed to the attenuated virulence phenotype observed in the animal model.

Growth studies showed that the *chtDE* or *srt* mutants were able to utilize the commercially prepared human Hb which was most likely in the form of metHb, freshly prepared human HbO_2_ and FeCl_3_ as the sole iron source at similar levels to the wild-type (Fig 6). These results indicate that heme acquisition by the Cht system might be required but is not essential for the growth of JIR325 under these conditions. This result is supported by studies in *S*. *aureus* and *L*. *monocytogenes* where the deletion of several *isd* genes also did not produce significant growth defects in the presence of commercial heme or Hb products as the sole iron source [83, 89, 90]. However, the results of these studies are in contrast to those which showed that several *isd* genes were essential for heme utilization by *S*. *aureus* when freshly prepared human Hb was provided as the sole iron source [57, 70].

Functional redundancies between iron acquisition systems have been reported in several organisms, where strains containing mutations in two different iron transport systems showed significant defects in utilizing heme/iron for its growth as compared to single mutants of an iron transport system [64, 91]. *C*. *perfringens* JIR325 contains seven putative iron uptake systems [27], thus deficiencies in Hb utilization of the *cht* mutants produced in this study may have been compensated by one or more of the other putative heme/iron acquisition systems found within this strain.

In summary, this study has demonstrated that the *C*. *perfringens cht* locus is expressed under iron-limited conditions and that the NEAT proteins, ChtD and ChtE can bind heme. The *chtD*, *chtE* and *srt* genes of the *cht* locus are most likely required but are not essential for FeCl_3_ or Hb utilization, and similarly do not appear to be essential for the growth and virulence of *C*. *perfringens* strain JIR325. Human Hb does not appear to be the major source of iron for *C*. *perfringens* strain JIR325, and as shown in other systems, one or more of the other putative heme/iron acquisition systems may have compensated for the deficiencies in Hb utilization of the *cht* mutants produced in this study.

## Supporting Information

S1 Fig Alignment of the NEAT domains of *C*. *perfringens* strain 13 to the NEAT or heme-binding domains of other Gram-positive bacteria. (TIF)

S1 Table Primers used in this study. (TIF)
