# Supplementary figures and images for: The NEAT Domain-Containing Proteins of Clostridium perfringens Bind Heme
Source: PLoS One. 2016 Sep 16;11(9):e0162981. doi: 10.1371/journal.pone.0162981 (PMC5026354; doi:10.1371/journal.pone.0162981)

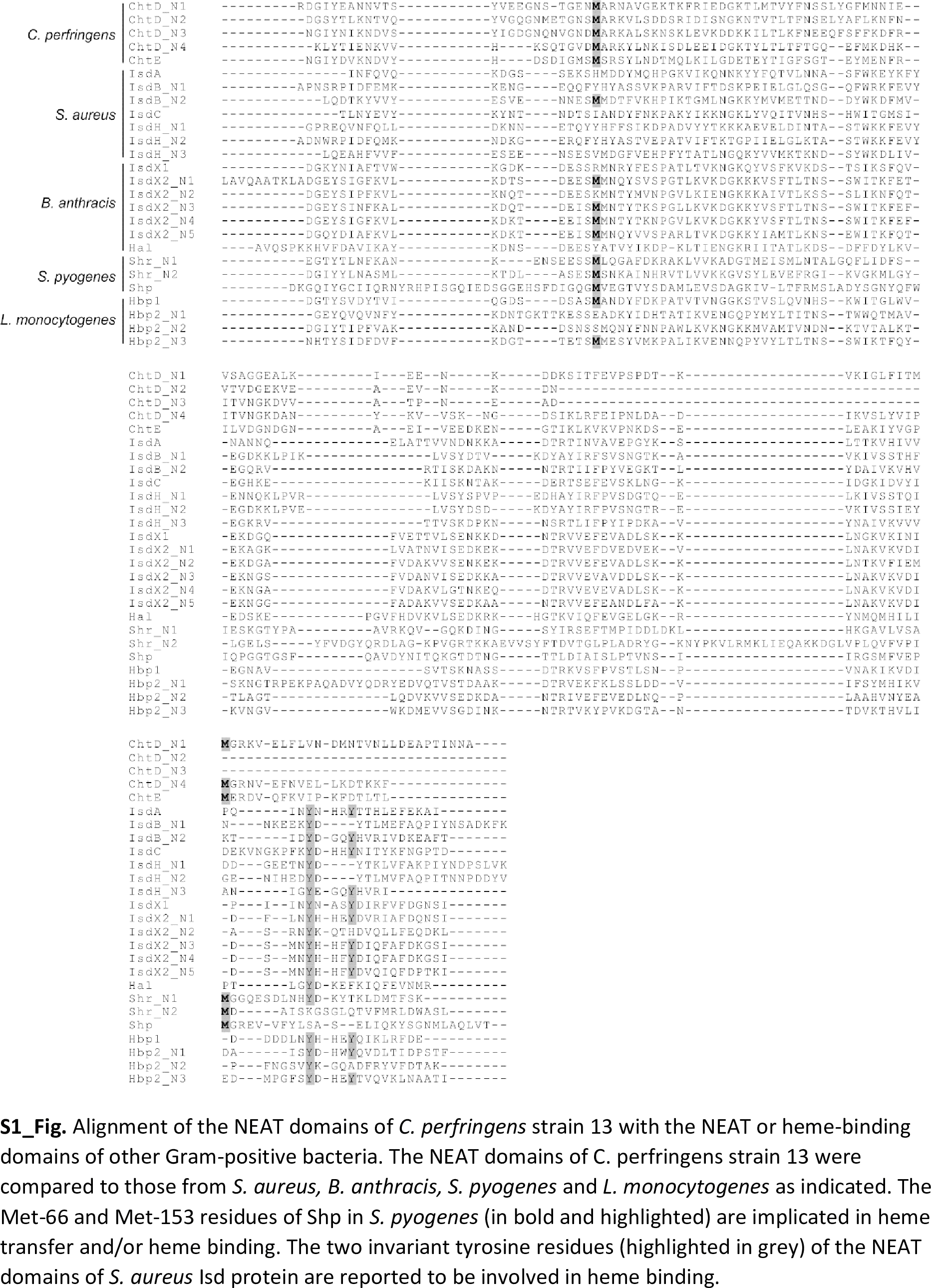

Supplement: S1 Fig — (TIF) [file pone.0162981.s001.tif]

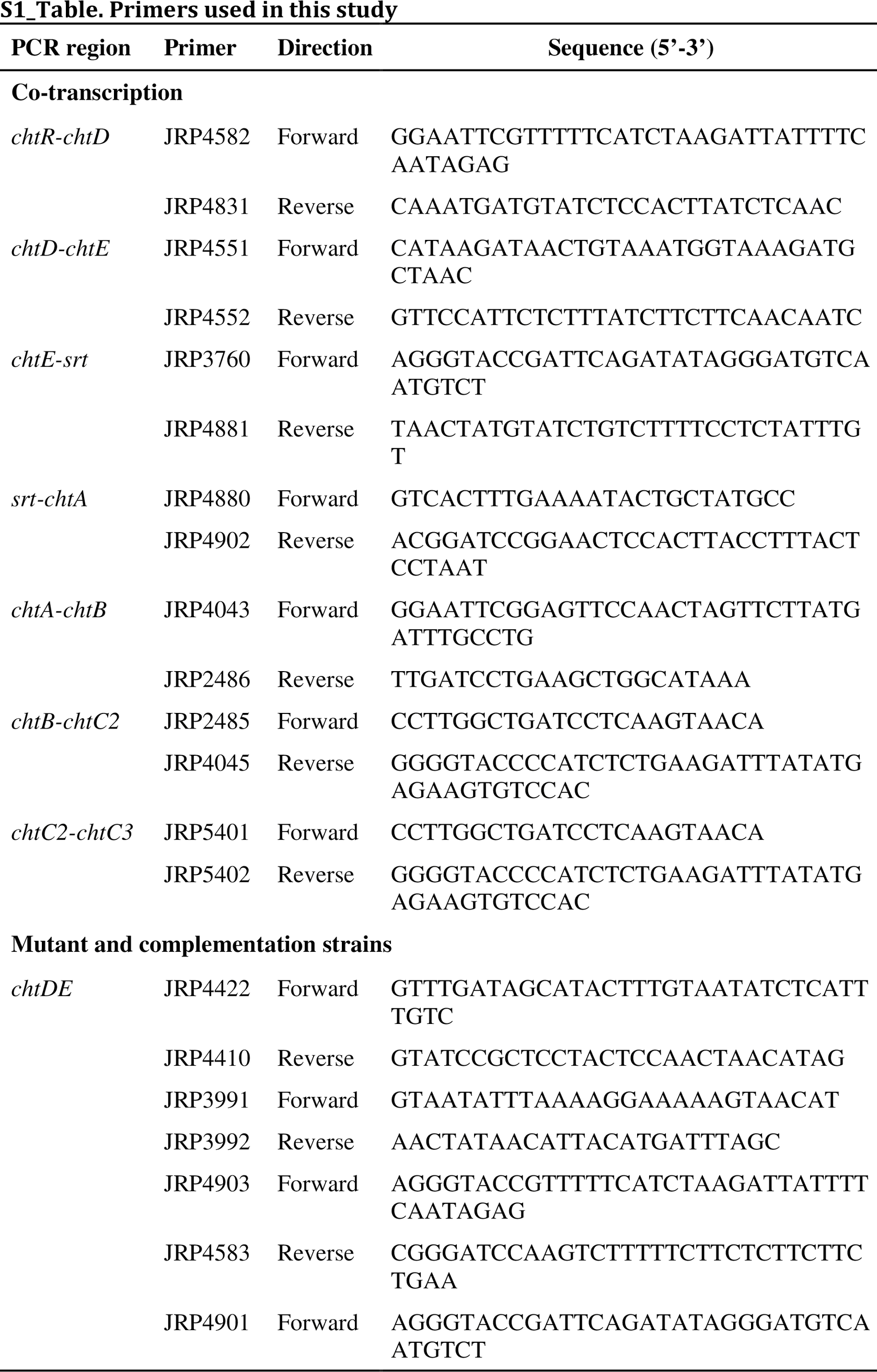

Supplement: S1 Table — (TIF) [file pone.0162981.s002.tif]
